# An Understanding of the Drivers of Infectious Diseases in the Modern World Can Aid Early Control of Future Pandemics

**DOI:** 10.3390/pharmacy9040181

**Published:** 2021-11-03

**Authors:** Taiwo Opeyemi Aremu, Oluwatosin Esther Oluwole, Kehinde Oluwatosin Adeyinka

**Affiliations:** 1 Department of Pharmaceutical Care & Health Systems (PCHS), College of Pharmacy, University of Minnesota, 308 Harvard Street SE, Minneapolis, MN 55455, USA; 2 Division of Environmental Health Sciences, School of Public Health, University of Minnesota, 420 Delaware Street SE, Minneapolis, MN 55455, USA; 3 Division of Epidemiology & Community Health, School of Public Health, University of Minnesota, 300 West Bank Office Building, 1300 S. 2nd Street, Minneapolis, MN 55454, USA; oluwo006@umn.edu; 4 Department of Radiation Oncology, University College Hospital (UCH), Ibadan 200248, Oyo State, Nigeria; Kehinde.adeyinka@uch-ibadan.org.ng

**Keywords:** infectious diseases, outbreaks and pandemics, antimicrobial resistance, vaccines and vaccine hesitancy, pharmacists

## Abstract

Infectious diseases have been a significant challenge to health and wellbeing from ancient times, with substantial economic implications globally. Despite the advent of technology, infectious diseases continue to affect people of various social statuses and across geographical locations. Understanding some of the drivers of infectious diseases, antimicrobial resistance, vaccination, and vaccine hesitancy is a step towards thriving in the modern world, achieving fewer morbidities and mortalities, and adequately controlling future pandemics. Pharmacists are strategically placed as healthcare team members to promote early disease control through health education, advocacy, cross-professional and specialty collaboration, communal trust-building, research, and global unity. Not forgetting that infectious diseases in the modern world are about people and science, credible crisis communication during the early phases of disease outbreaks paves the way for well-informed guidance globally.

## 1. Introduction

Health and wellbeing concerns across the globe have continued to grow over the past century. The sustainable development goals (SDGs) were designed to provide what is required to thrive in the contemporary world, including better access to healthy food, clean water, sanitation, education, healthcare, etc. As the world’s population increases, so do forest encroachment, urbanization, international travel, migration, etc. These events consequently increase the world’s challenges. Among these consequences are infectious diseases. Although infectious diseases have been in existence for as long as humans have, the world continues to experience a newer but similar dimension of outbreaks that threatens human health. Infectious diseases and outbreaks have implications for health and wellbeing, including adverse morbidity and mortality in infected persons. The economic impact of infectious disease outbreaks on individuals and society is enormous. In recent years, the world has experienced different epidemics; examples include the COVID-19, Zika, Ebola, Swine flu (H1N1), severe acute respiratory syndrome (SARS), etc. The trends of infectious diseases in the modern world show that infections affect people, cut across geographical locations and boundaries, and transcend time.

### 1.1. People

Organisms such as bacteria, viruses, fungi, or parasites cause infectious diseases [1]. Some are transmitted to humans from humans (e.g., HIV, Chlamydia, bacterial vaginosis, etc.), insects (e.g., malaria from mosquitoes) [2], or animals (e.g., avian flu from birds) [3]. Humans can get infected through direct contact, such as from person to person (through touch, kiss, coughs, sneezes, sexual contact, etc.), animals to persons (through bites, scratches, handling of waste, etc.), and mother to unborn child (through the placenta, e.g., toxoplasmosis, rubella, cytomegalovirus, herpes simplex, etc.) [4]. Humans can also get infected through indirect contact, insect bites (e.g., Lyme disease from ticks, the West Nile virus, etc.), and water and food contamination (e.g., typhoid fever from Salmonella typhi, cholera from Vibrio cholerae, shigellosis from Shigella, etc.) [4].

Understanding how risk, evaluation behaviors, prevention behaviors, actions, and inactions affect human response to infectious diseases is important. Phenomena such as antimicrobial resistance, vaccination, and vaccine hesitancy are some factors that drive infectious diseases in the modern world.

#### 1.1.1. Antimicrobial Resistance

According to the World Health Organization (WHO), antimicrobial resistance (AMR) occurs when bacteria, viruses, fungi, and parasites change over time and no longer respond to medication (antibacterial/antibiotics, antiviral, antifungal, and antiparasitic) [5]. AMR is mostly driven by consumption (misuse and overuse). Bacteria, viruses, fungi, and parasites change to become unresponsive to medication, hence becoming more challenging to treat and increasing disease spread, severe illnesses, and death [5,6]. Other factors include inadequate access to quality, affordable medication, vaccines, and diagnostics; poor disease control in healthcare centers and farms; lack of knowledge/awareness; lack of regulations, etc. Aside from the abuse of antimicrobial medications by patients, general practitioners are thought to be culprits in the non-rational use of antibiotics, especially when they prescribe antibiotics before bacterial infection confirmatory test results are ready [7,8]. Community pharmacies in low-resource countries where antibiotics are readily available over the counter due to inadequate regulation are likewise culprits. The overuse of antimicrobial supplements in animal feeds by veterinarians has also led to a rise in antibacterial resistance in humans who have contact with or consume animal products [9]. Evidence shows that the use of antibiotics during the early months of the COVID-19 pandemic was not associated with the presence of bacterial infection, suggesting the irrational use of antibiotics in COVID-19 patients globally [10]. This has implications for a possible increase in AMR in the future of humanity.

Antimicrobial resistance is a threat to global health, with extensive health and economic implications. About 700,000 people die from bacterial-resistant infections globally every year [11]. The Centers for Disease Control and Prevention (CDC) reports more than 2.8 million cases of AMR infection and more than 35,000 deaths from AMR infections each year in the United States [12]. When AMR occurs, we may need new and more potent microbial medications. Economically, the development of antimicrobial medicines is expensive, costing over $1.7 Billion to bring a new antibiotic drug to market [13].

#### 1.1.2. Vaccination and Vaccine Hesitancy

Vaccines have provided vital contributions to the global reduction of morbidity and mortality. Vaccine-preventable childhood infectious diseases such as tetanus, pertussis, measles, diphtheria, poliomyelitis, meningitis-encephalitis, acute respiratory infections, etc., and their resulting morbidity and mortality have cumulatively decreased globally [14]. Although vaccines are meant to prevent or treat infectious diseases and their complications through the stimulation of the immune system, some major hindrances to vaccination, such as vaccine nationalism, vaccine inequity, and vaccine hesitancy, still prevail [15].

Vaccine nationalism, which refers to “taking care of our own first”, has implications for pandemic responses, especially with the emergence of infectious disease variants that may crop up from developing countries, threatening everyone else. This is evident with the COVID-19 pandemic, where new variants with the potentials of evading vaccines have sprung up and are still springing up from the seemingly neglected regions of the world. If allowed to spread uncontrollably, the latest COVID-19 variants may result in more deaths. The WHO Director-General, Tedros Adhanom Ghebreyesus, once said that “vaccine equity is the challenge of our time, and we are failing”. Despite having the tools to end the COVID-19 pandemic, the world faces a resurgence, partly caused by the dramatic inequity of vaccine coverage [16].

Vaccine hesitancy, a global health threat, refers to the refusal to vaccinate despite vaccine availability [17]. People choose not to vaccinate due to complex reasons, including varied ideologies and beliefs, complacency, the inconvenience of accessing vaccines, and a lack of trust [17,18,19,20]. With a vast audience, the media has historically been used as a channel to disseminate misinformation and conspiracy theories [18]. History is replete with examples of countries where vaccine hesitancy has thrived due to leaders’ inadequate political will and incorrect information dissemination through fake news, propaganda, and conspiracy theories.

### 1.2. Geographical Locations and Boundaries

Infectious disease in the modern world has no regard for geographical boundaries. History is replete with examples of clusters of outbreaks that eventually became the struggle of many world regions. Irrespective of social and economic status and privilege, the world is a small village; everyone can be subjected to the same exposure but with varying magnitudes depending on human behavior. However, irrespective of location, everyone everywhere can be a recipient of localized spillage. For example, the Japanese encephalitis outbreak in Asia and the western pacific started in southern Asia before spreading to other regions and across borders to become endemic [21,22].

Sociocultural practices in certain regions can be a precursor to outbreaks. In addition, population growth, forest encroachment, and direct interaction with wildlife, e.g., bushmeat consumption, can contribute to the spread of infectious diseases like the Ebola virus [23,24]. The COVID-19 pandemic and how it started is a testament to this narrative. If we choose to ignore what happens in the forgotten corners of this small world, it may be to our peril.

### 1.3. Time

Every living thing on earth has a time tag. In the events that occur during human existence, such as infectious diseases, time is as an essential factor. From its inception to its spread, time is a critical factor of infectious disease. We live in a world where a delay in response to any natural or human-made disaster can result in the loss of lives and resources. Due to the timely response to the Ebola virus outbreak in Nigeria in 2014, the WHO declared Nigeria Ebola-free after three months [25].

The U.S. has been able to control clusters of foodborne disease outbreaks due to the timely reporting of individuals to the hospital and the timely reporting of health officials to state health departments. The timely response of the U.S. Food and Drug Administration (FDA) and the CDC to these reports of outbreak clusters and the issuance of food product recalls by the companies involved have been useful in controlling foodborne disease outbreaks in the U.S. The value of timely responses to outbreaks’ management cannot be over-emphasized.

How the COVID-19 pandemic started and its overwhelming effect on the world is proof that a delay in the response to the spillage of infective agents anywhere in the world can become a global disaster.

## 2. The Role of Pharmacists in the Control of Infectious Diseases in the Modern World

### 2.1. Health Education

To mitigate the health impact of AMR on people, pharmacists, being stewards of medicines and specialized in the preparation, usage, storage, preservation, and provision of drug-related information to the public, are in a good position to encourage the appropriate use of antibiotics, referred to as antimicrobial stewardship [26]. As integral members of the healthcare team, pharmacists are strategically placed to advance the understanding of antibiotics and their judicious use among consumers, a phenomenon known as consumer education [27]. To further address antimicrobial resistance adequately, we can leverage our ability to educate present and future prescribers through collaboration, conference presentations, publications in clinical journals, etc., to cautiously prescribe antimicrobial medications [28]. All prescribers and members of the care team (clinicians, nurses, pharmacists, etc.) should be stewards of proper antimicrobial medication use and should educate their patients to use them appropriately. We can also reduce antibiotic consumption through the One Health antimicrobial stewardship practice, considering how antibiotic use in animals can impact the health of humans. As an alternative antimicrobial strategy, the use of prebiotics and probiotics are only indicated in specific conditions following evidence-based recommendations, and as such should not be routinely encouraged [29].

Pharmacists, as healthcare professionals, are trusted advisors and influencers of vaccination decisions and must be resolute about reaching the unreached with the information that may encourage them to make well-informed decisions. We should use evidence-based messages to refute misinformation and conspiracy theories on social media tactically. We must be equally careful not to over reassure; we must be transparent in proclaiming uncertainty and admitting to our mistakes because these make for good leadership during a crisis and help us all cope [30].

The latest COVID-19 pandemic and the established safety guidelines exemplify how we can apply health education to control infectious diseases in the modern world. As trusted advisors, community pharmacists, and other health professionals may help prevent the spread of COVID-19 using visual presentations in the form of posters to depict the need for proper hand hygiene, social distancing, the wearing of face-coverings/masks, and the COVID-19 vaccine [31].

### 2.2. Advocacy

Because we understand that we are dealing with humans as the recipients of our actions and inactions, this should spur us into action to advocate the “humanitarian” sharing of vaccines across regions. Community pharmacists can achieve this by using professional bodies and associations as platforms to suggest policy-based solutions to local and global health challenges. In addition, to capture the undocumented persons in our vaccine coverage in the developed countries, we can propose a limit to the personal information required to get access to vaccinations.

An old proverb says, “where there is a will, there is a way”. Therefore, to make impactful improvements, we should be willing to lead the way in supporting value-based change. We can also inspire the applicable local, federal, and international agencies to provide economic incentives for early reporting of suspected exposures and outbreaks because humans respond to financial incentives by their very nature [32]. 

### 2.3. Partnership and Global Unity

We can actively encourage partnership between private and public organizations to develop new technologies such as a rapid microscopy sensitivity and culture (MCS) method for the timely detection of organisms in people’s systems to inform the prescription of antibiotics. Because of the diverse population that we serve, our ability to build collaborations with community and faith-based organizations’ leaders can help us reach members of these communities.

Joint efforts by the WHO, the United States Centers for Disease and Prevention (CDC), Médecins Sans Frontières (MSF), United Nations, and national and local government agencies helped control the Ebola outbreak in West Africa [25]. The partnership helped provide facilities for outbreak investigation, risk assessment, contact tracing, and clinical care [25]. This shows that we can achieve so much irrespective of geographic boundaries when we work together towards a common goal, improving global health.

The ability to work with other professionals in healthcare, the industry, and elsewhere, irrespective of geographical location or boundaries, may provide the tools to combat future pandemics more effectively. To combat the current COVID-19 pandemic, the WHO Director-General, Tedros Adhanom Ghebreyesus, said, “we cannot defeat the coronavirus one country at a time; we can only do it with a coordinated global effort, based on the principles of solidarity, equity, and sharing”. The emergence of the coronavirus and its variants with its devastating effect that threatens the available vaccines’ efficacy proves that unity of purpose is more needed than ever [33]. Indeed, more connects us than separates us.

### 2.4. Research and Development

The active involvement of pharmacists in the innovative development of rapid diagnostic testing materials such as rapid MCS kits and the use of community pharmacies in the testing of these deliverables is a step towards a breakthrough. The subsequent availability of these deliverables in community pharmacies, following their statutory approval, may drive well-informed prescribing and mitigate the indiscriminate administration of antimicrobial agents in community pharmacies, especially in low-resource countries. 

### 2.5. Build Trust

We should scale up our messaging with credible messengers to decrease the vaccine hesitancy in our society, understanding that trust is the golden glue [34]. Because our collective safety is everyone’s business, it will serve us well in the long run if we play our part in ways possible towards trust-building. In reality, “no one can do everything, but everyone can do something [35]”.

### 2.6. Seek opportunity, Be Intentional

As professionals with the privilege of setting up outlets in the community, community pharmacies can be used as platforms to encourage the early and detailed reporting of outbreaks, especially when there are suspicions of exposures or the presence of symptoms. As potential political leaders, it may be worthwhile to remember that solid political commitments to public health may help drive the early control of disease outbreaks in the modern world.

## 3. Conclusions

Infectious disease is a threat to national and global security, hence a need to pay attention and fight it early anywhere it is found as our common enemy. Irrespective of boundaries, people suffer and die if we neglect to respond and help fight infectious diseases anywhere in the world. As stewards of medication, pharmacists have the unique ability to mitigate some of the factors that drive the spread of infectious diseases. The willingness to chart the path of unity in research and innovative development, knowledge sharing with other healthcare team members, community health education, trust-building, and advocacy are recipes for early control of future outbreaks and pandemics. We must collectively work through the pharmacist professional bodies to persistently demand that political and public health leaders, journalists, and other commentators imbibe the principles of crisis communication during the early phases of disease outbreaks. Finally, we should remember that infectious diseases in the modern world are all about people and science.

## Data Availability

Not applicable.

## Abbreviations

SDG (sustainable development goals), AMR (antimicrobial resistance), MCS (microscopy sensitivity and culture), HIV (human immunodeficiency virus).
